# Hemorrhagic Complications of Paracentesis: Aberrant Anatomy Versus Aberrant Technique - A Fatal Case of Abdominal Hemoperitoneum

**DOI:** 10.7759/cureus.8827

**Published:** 2020-06-25

**Authors:** Andrew C Berry

**Affiliations:** 1 Gastroenterology, Larkin Community Hospital, South Miami, USA

**Keywords:** paracentesis, liver cirrhosis, liver disease, ascites, radiology, bleeding, hemoperitoneum

## Abstract

Large-volume paracentesis carries roughly a 1% risk of overall complications. Hemorrhagic complications are classified as abdominal wall hematomas, pseudoaneurysms, and hemoperitoneum. Severe hemorrhage is rare (<0.2%), with death following this complication seen in <0.02% of cases. We present a fatal case of an ultrasound-guided paracentesis leading to subsequent hemoperitoneum from an aberrant intercostal artery, causing hemorrhagic shock and death. A 47-year-old black male with decompensated alcoholic cirrhosis, model for end-stage liver disease (MELD) score of 22, and Child-Pugh class C presented with a distended abdomen, international normalized ratio (INR) 1.9, and hemoglobin 9.6 g/dL. An ultrasound-guided therapeutic paracentesis was performed in the right lower quadrant with 50 mL intravenous albumin given after 4 L of uncomplicated ascitic fluid removal. The patient became hypotensive, tachycardic, and placed on pressor support medication within 12 hours after the procedure. After a complex hospital course, the patient passed away on hospital day 10 after multisystem organ failure. The patient was found to have an aberrant intercostal artery bleed secondary to the paracentesis procedure causing an abdominal hemoperitoneum.

## Introduction

Many patients with decompensated liver disease require diuretic dose optimization and the occasional abdominal paracentesis for volume removal or diagnostic purposes [1]. A paracentesis is regarded as a very safe procedure, albeit solely performed via traditional anatomical landmarks or the much more frequent ultrasound-guided approach [2]. Large-volume abdominal paracentesis carries roughly a 1% risk of overall complications, which may include leakage of ascitic fluid, infection, bleeding, or bowel perforation [2-5]. Hemorrhagic complications are very uncommon, and are classified as abdominal wall hematomas, pseudoaneurysms, and hemoperitoneum. Severe hemorrhage is rare (<0.2%), with death following this complication seen in <0.02% of cases [2-8]. Volume shifts after therapeutic paracentesis can mask an initial minor drop in hemoglobin, and it remains prudent to carefully observe any post-procedural patient in case clinical changes develop. We present a case of an ultrasound-guided paracentesis leading to subsequent hemoperitoneum from an aberrant right intercostal artery, causing hemorrhagic shock and death.

This work has been previously published as a limited conference abstract proceeding (Conference: Berry AC, Ludvik N, Winburn R, Bolling C, Schultz J, Henderson PK. Hemorrhagic Complications of Paracentesis: Aberrant Anatomy versus Aberrant Technique -What is the Culprit? 2017 American College of Gastroenterology (ACG) Annual Scientific Meeting and Postgraduate Course - World Congress of Gastroenterology (WCOG); October 13-18, 2017).

## Case presentation

A 47-year-old black male with decompensated alcoholic cirrhosis, model for end-stage liver disease (MELD) score of 22, and Child-Pugh class C presented with a distended abdomen, international normalized ratio (INR) 1.9, and hemoglobin 9.6 g/dL to a large academic teaching medical center. He denied any fever or significant abdominal pain. He did have prior abdominal paracentesis performed without any complications over the past calendar year. In the emergency room, an ultrasound-guided therapeutic paracentesis was performed in the right lower quadrant with 50 mL 25% intravenous albumin given after 4 L removal, all by a trained clinical staff member. The patient became hypotensive, tachycardic, and placed on pressor support medications within 12 hours after procedure. No spontaneous bacterial peritonitis was found on fluid analysis. Hemoglobin overtly dropped to 3.3 g/dL and INR rose to 7.

**Figure 1:**
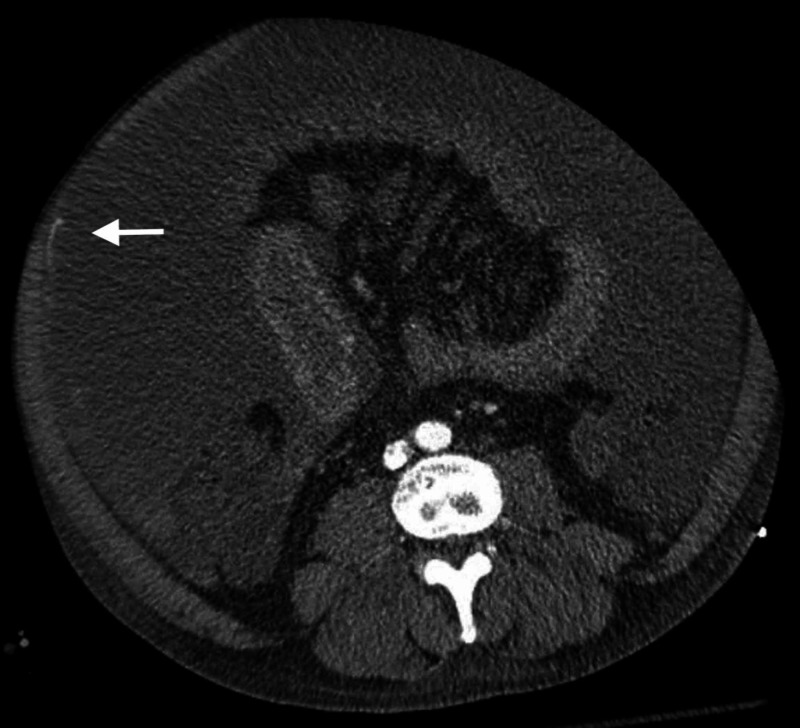
Axial CT angiography of the abdomen with diffuse abdominal ascites with a linear hyperattenuation adjacent to the right lateral abdominal wall (arrow). Subtle dependent densities are seen adjacent to the right posterolateral abdominal wall, layering within the ascites fluid.

Axial CT angiography of the abdomen depicts overt ascites with a linear hyperattenuation adjacent to the right lateral abdominal wall, with dependent densities layering within the fluid (Figure 1).

**Figure 2:**
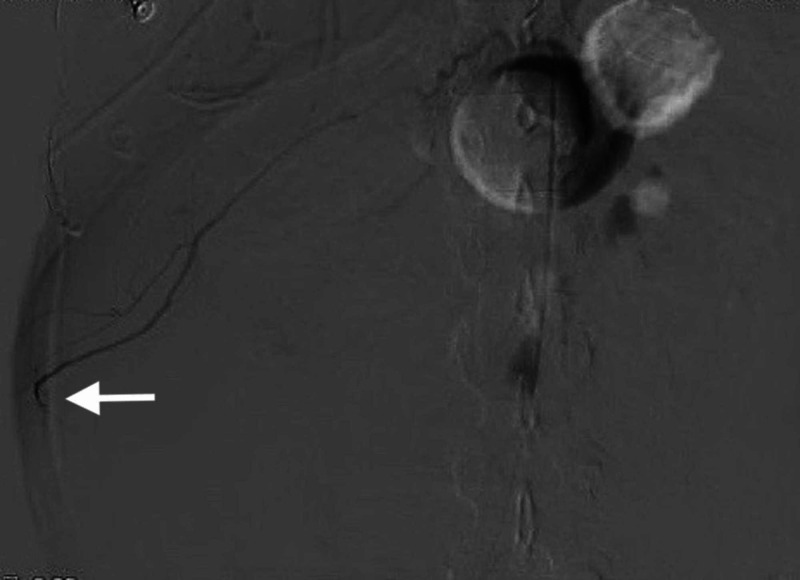
Digital subtraction angiography image at the level of the right upper abdomen with contrast extravasation at level of a right intercostal artery (arrow).

Further, digital subtraction angiography is shown with extravasation of contrast from a right intercostal artery (Figure 2). 

**Figure 3:**
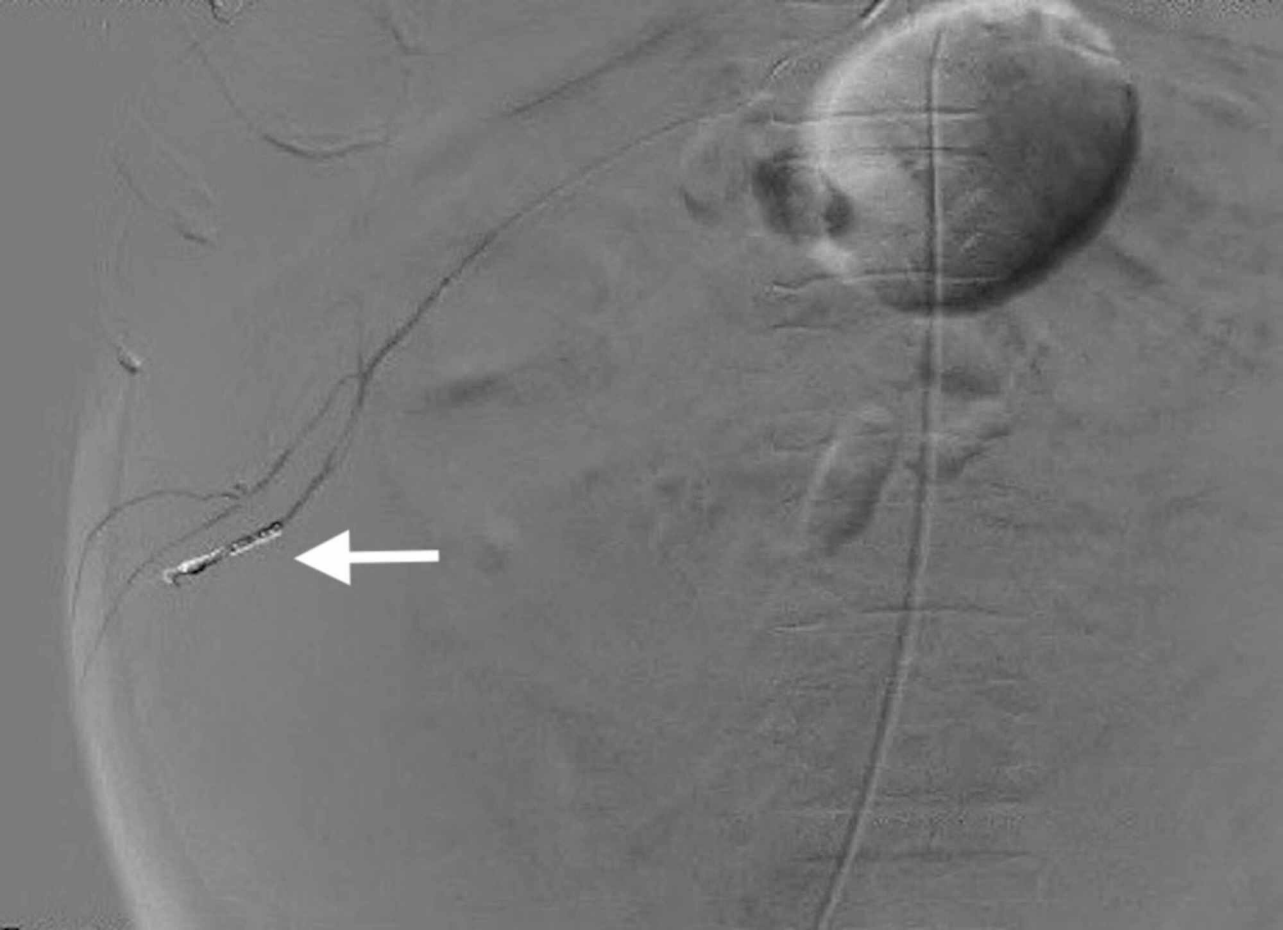
Axial CT angiography of the abdomen with contrast filling the selected right intercostal artery following catheter-directed placement of embolization coils (arrow) and no further contrast extravasation.

Following catheter-directed placement of embolization coils by the interventional radiology (IR) team, contrast is seen filling the selected right intercostal artery, with non-visualization of flow distal to the coils (Figure 3), confirming hemostasis. 

**Figure 4:**
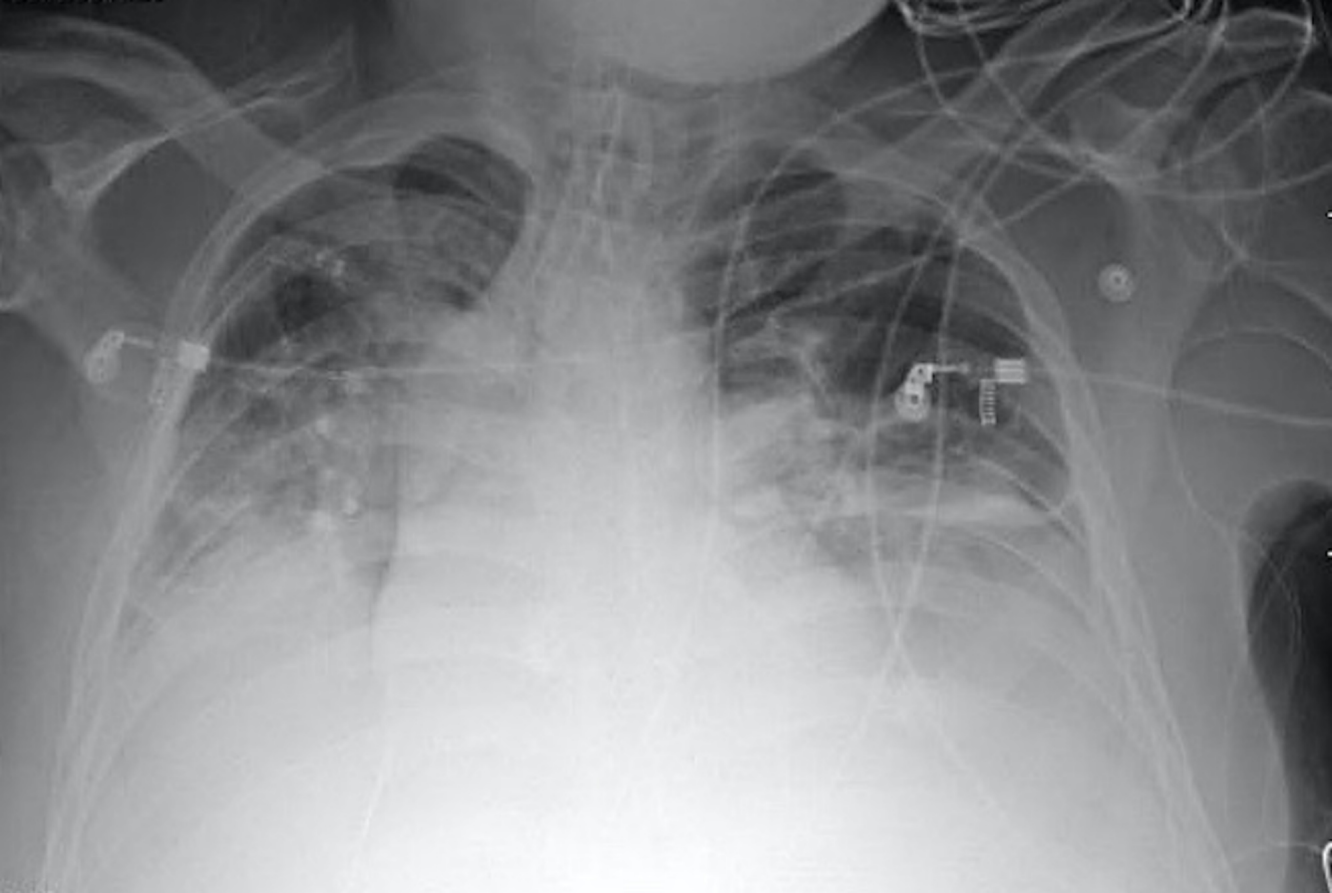
Posterior-anterior view of the chest X-ray showing bilateral fluid densities in the lungs, indicating acute respiratory distress syndrome.

IR intervention was performed rather quickly from time of noted clinical change, within six to eight hours. Ultimately, the patient was intubated early the next morning following IR intervention, and developed acute respiratory distress syndrome as seen on the chest X-ray (Figure 4). After receiving numerous blood products and hemodynamic support, the patient passed away on hospital day 10 due to multisystem organ failure and family withdrawal of care. 

## Discussion

An abdominal paracentesis is a safe procedure with minimal complication risks; however, in some cases like ours, routine diagnostic or therapeutic paracentesis may lead to rare hemorrhagic complications and even death [2]. When major bleeding is present, IR-guided intervention has lower 30-day mortality than surgical intervention, with combined 30-day mortality around 40% [2]. It is prudent to remember that this decompensated patient population already carries a high risk of mortality, as dictated by their MELD score. Our patient's demise amid successful embolization of an intercostal artery bleed likely resulted from subsequent hemodynamic and circulatory response, as the patient required multiple pressor support medications and copious volume resuscitation. Decompensated liver disease patients with subsequent hemodynamic and circulatory flux carry an elevated risk of adverse outcomes. 

Routine paracentesis performed by trained general internal medicine, emergency medicine, or specialist staff is considered satisfactory, safe, and cost-effective. A rare and fatal complication from an aberrant anatomical derivative should not hinder future procedures performed by hospital non-radiologic staff. In fact, lack of delay in performing paracentesis by non-radiologic staff may actually be more efficacious for patient length of hospitalization and survival. In one retrospective study, IR referrals for paracentesis were significantly associated with 1.86 additional hospital days, higher requirement of platelet and fresh-frozen plasma transfusions, and higher rate of transfer to the intensive care unit (ICU) after undergoing paracentesis [3]. Further studies have shown that patients are quite satisfied with bedside procedures, and even those performed by trainees under supervision. In fact, nearly all patients were satisfied or very satisfied with the overall experience (100%) and expertise (95%) of involved physicians, along with the procedure duration (88%) and improvement in symptoms (89%) [9].

All patients with decompensated ascites who present to the hospital with any clinical complaint should at least undergo a timely diagnostic paracentesis per the American Association for the Study of Liver Diseases (AASLD) clinical guidelines [1]. Trained physicians must optimize pre- and post-procedural paracentesis safety checklists to develop system-wide protocols to help minimize any procedural complications. In fact, studies have shown that physicians utilizing a standardized procedure checklist and equipment kit demonstrated noted improvements in documentation rates, and most importantly, a significant decline in post-paracentesis complications [10].

## Conclusions

Overall, complication rates are low following paracentesis in chronic decompensated liver disease patients; however, it remains prudent to monitor any clinical change in the peri-procedural period and act in a timely, multidisciplinary manner when complications arise. It remains advantageous to understand possible complications prior to any procedure, but to not let a nominal risk hinder timely and adequate clinical care in the context of this vulnerable decompensated liver disease patient population.

## References

[1] Runyon BA (2013). Introduction to the revised American Association for the study of liver diseases practice guideline management of adult patients with ascites due to cirrhosis 2012. Hepatology.

[2] Sharzehi K, Jain V, Naveed A, Schreibman I (2014). Hemorrhagic complications of paracentesis: a systematic review of the literature. Gastroenterol Res Pract.

[3] Barsuk JH, Cohen ER, Feinglass J, McGaghie WC, Wayne DB (2013). Clinical outcomes after bedside and interventional radiology paracentesis procedures. Am J Med.

[4] Guzman Rojas P, Sachdeva R, Blonski W (2019). Delayed retroperitoneal hemorrhage as a complication of large-volume paracentesis. Cureus.

[5] Lin S, Wang M, Zhu Y (2015). Hemorrhagic complications following abdominal paracentesis in acute on chronic liver failure: a propensity score analysis. Medicine.

[6] Qureshi WA, Harshfield D, Shah H, Netchvolodoff C, Banerjee B (1992). An unusual complication of paracentesis. Am J Gastroenterol.

[7] MacDonald GR (1951). Exsanguination, a complication of paracentesis abdominis. Treat Serv Bull.

[8] Serbin RA (1956). Fatal hemorrhage from paracentesis; a case of Cruveilhier Baumgarten syndrome. Gastroenterology.

[9] Mourad M, Auerbach AD, Maselli J, Sliwka D (2011). Patient satisfaction with a hospitalist procedure service: Is bedside: Is bedside procedure teaching reassuring to patients?. J Hosp Med.

[10] Fyson J, Chapman L, Tatton M, Raos Z (2018). Abdominal paracentesis: use of a standardised procedure checklist and equipment kit improves procedural quality and reduces complications. Intern Med J.

